# Association between combined healthy lifestyles and infertility: a cross-sectional study in US reproductive-aged women

**DOI:** 10.1186/s12889-025-21395-2

**Published:** 2025-01-14

**Authors:** Xiaofeng Ye, Xiaoxia Song, Sihang Zhou, Guoqing Chen, Liping Wang

**Affiliations:** 1 Reproductive Medicine Centre, The First Affiliated Hospital of Shenzhen University, Shenzhen Second People’s Hospital, Guangdong Key Laboratory for Biomedical Measurements and Ultrasound Imaging, National-Regional Key Technology Engineering Laboratory for Medical Ultrasound, School of Biomedical Engineering, Shenzhen University Medical School, Shenzhen, 518060 China; 2 Reproductive Medicine Centre, The First Affiliated Hospital of Shenzhen University, Shenzhen Second People’s Hospital, Shenzhen, 518035 China; 3 Health Science Center, Shenzhen University, Shenzhen, 518060 China

**Keywords:** Combined healthy lifestyles, National Health and Nutrition Examination Survey (NHANES), Infertility, Smoking, Waist circumference, Alcohol drinking, Healthy diet, Physical activity

## Abstract

**Background:**

Infertility is a widespread problem for couples worldwide, and lifestyle factors are the cornerstone of infertility prevention. This research seeks to explore the association between combined healthy lifestyles and infertility risk among women of reproductive age.

**Methods:**

This study analyzed data from the National Health and Nutrition Examination Survey (NHANES, 2013–2018), concentrating on 2,154 women aged 18 to 44. A healthy lifestyle score was created based on healthy diet (top two-fifths of the Healthy Eating Index-2015 score), low-to-moderate alcohol drinking (1–14 g/day), optimal waist circumference (less than 80 cm), adequate physical activity (at least 150 min of moderate-to-vigorous leisure-time exercise per week), and current nonsmoking. Weighted logistic regression analysis was applied to assess the link between healthy lifestyle scores and the risk of infertility, adjusting for potential confounders.

**Results:**

After adjusting for potential confounders, women exhibiting 4 to 5 healthy lifestyle factors demonstrated a 59% decrease in the likelihood of infertility (OR: 0.41, 95% CI: 0.23–0.76) relative to those with 0 to 1 healthy lifestyle factors. Additionally, each increment in healthy lifestyle factors corresponded to a 21% decrease in infertility risk (OR: 0.79, 95% CI: 0.68–0.92). Analysis of subgroups indicated that the inverse association was more pronounced in females younger than 30. Additionally, optimal waist circumference is the foremost factor contributing to this inverse relationship.

**Conclusion:**

Adhering to healthy lifestyles significantly lowers the likelihood of infertility among reproductive-aged women. Public health initiatives could consider enhancing access to healthy diets, physical activity, and resources to reduce alcohol consumption and smoking. Further research is required to clarify their relationship and the underlying mechanisms.

**Supplementary Information:**

The online version contains supplementary material available at 10.1186/s12889-025-21395-2.

## Introduction

Infertility is a widespread problem for couples worldwide [1]. A recent World Health Organization (WHO) report shows that around 17% of individuals will encounter infertility [2]. Infertility has an impact on the emotional, physical, and social well-being of both individuals and families [3, 4]. Assisted reproductive technology (ART) is an effective infertility treatment, but it comes with high costs and potential treatment-related medical risks [5, 6]. Consequently, it is paramount to pinpoint modifiable risk factors and develop cost-effective approaches to mitigate these societal and familial pressures.

Numerous risk factors contribute to the rising incidence of infertility, with age being a critical determinant [7–9]. Notably, the age of 30 represents a significant threshold for female fertility. Women under 30 enjoy an 85% chance of conceiving within one year, while this likelihood declines to 75% by the age of 30 [10, 11]. This decline is fundamentally linked to the detrimental effects of aging on ovarian reserve and oocyte quality [9, 12]. Additionally, obesity among women has been shown to compromise fertility, primarily through its adverse effects on ovulation and endometrial receptivity [13]. Many metrics assess obesity levels in women, with waist circumference being strongly correlated with infertility risk, as it reflects both obesity and visceral fat accumulation [14, 15]. Additionally, an imbalanced diet, alcohol intake, smoking, and insufficient physical activity can also contribute to infertility [16–18].

Despite these established associations, existing research primarily utilizes logistic regression to analyze individual risk factors in isolation. Moreover, much of the current literature is limited by its focus on homogeneous populations, frequently drawn from specific ethnic or geographic groups, with insufficient adjustments for confounding variables [8, 19, 20]. This limited approach may oversimplify the complex nature of infertility and overlook interactions between lifestyle factors that could affect their association with infertility.

Evidence regarding the combined impact of lifestyle factors on infertility is limited. The Nurses’ Health Study II cohort study found that the incidence of infertility diminished with a greater number of low-risk lifestyle factors; however, the analysis focused solely on ovulatory disorder infertility and considered only diet, physical activity, and weight control, neglecting additional lifestyle factors (e.g., alcohol drinking and smoking) [21]. Furthermore, the study’s exclusive reliance on a nursing population raises questions about the generalizability of its findings to the wider community.

To bridge the theoretical voids, we leveraged data from the National Health and Nutrition Examination Survey (NHANES) to conduct a comprehensive analysis of the relationships between combined healthy lifestyles and infertility. This analysis employed a healthy lifestyle score made up of five components: diet, physical activity, waist circumference, smoking, and alcohol consumption. This score captures interactions among these factors and enhances our understanding of how they collectively influence infertility.

## Methods

### Study population

The NHANES is a nationally representative program that evaluates the health and nutritional status of the general population in the United States (US). The Ethics Review Board of the National Center for Health Statistics (NCHS) approved the study and secured informed consent from all participants. We selected 3,517 non-pregnant females of reproductive age (18–44 years) from the NHANES 2013–2018 cycles for eligibility screening. After excluding 559 subjects with missing infertility data and 645 subjects with incomplete lifestyle information, 2,313 females remained. Furthermore, 159 participants with incomplete covariate data were removed from the analysis. The counts and percentages of missing data for the covariates are presented in Table S1. Ultimately, 2,154 females of reproductive age were included, with the eligibility screening process illustrated in Figure S1.

### Definition of infertility

Self-reported infertility was determined from participants’ responses to the question, “Have you ever attempted to become pregnant over a period of at least a year without becoming pregnant?” Individuals were categorized as infertile if they responded with “yes.”

### Construction of healthy lifestyle score

The healthy lifestyle score was constructed by tallying the quantity of healthy lifestyle factors, including current nonsmoking, adequate physical activity, low-to-moderate alcohol drinking, optimal waist circumference, and healthy diet, as per Zhang et al.‘s method [22, 23]. The healthy lifestyle score ranges from 0 to 5, with higher scores reflecting healthier lifestyle choices. The criteria for healthy lifestyle levels are presented in Table S2. According to the US dietary guideline, alcohol consumption of 1–14 g/day for women was deemed healthy levels [24]. Current nonsmoking was defined as a healthy level [25]. Healthy physical activity levels were established as engaging in moderate-to-vigorous leisure-time exercise for at least 150 min/week [22, 25]. The Healthy Eating Index-2015 (HEI-2015) was utilized to assess diet quality. Based on a previous study using the US NHANES data, a healthy diet was labeled as being in the top two-fifths of the HEI-2015 score [22, 25, 26]. The current study used waist circumference to assess obesity, with healthy levels set at less than 80 cm for women, under WHO recommendations [27, 28].

### Covariates

Marital status, race/ethnicity, education, family poverty-income ratio (PIR), diabetes, and hypertension were considered covariates in the analysis. Race/ethnicity includes non-Hispanic black, non-Hispanic white, Mexican American, and others [29]. Marital status was classified as married and others (divorced, widowed, separated, living with a partner, and never married). Self-reported education was grouped into three levels: below high school, above high school, and high school [30]. PIR was calculated by dividing income by the survey year’s poverty guidelines. Blood pressure readings were obtained by trained examiners utilizing mercury sphygmomanometers. Hypertension was labeled as either a diastolic blood pressure ≥ 90 mmHg, a systolic blood pressure ≥ 140 mmHg, active use of prescribed antihypertensive medication, or a hypertension diagnosis from a physician. Diabetes was classified based on any of the following criteria: glycated hemoglobin A1c (HbA1c) ≥ 6.5%, fasting plasma glucose (FPG) ≥ 126 mg/dL, a two-hour glucose level ≥ 200 mg/dL from an oral glucose tolerance test, usage of insulin or oral diabetes medications, or a physician’s self-reported diabetes diagnosis.

### Statistical analysis

Due to the limited number of subjects scoring 0 and 5, scores of 0 and 1 were merged, along with scores of 4 and 5 (Table 1). Quantitative variables were represented by weighted means and standard errors (SEs), and their means were compared using linear regression. Qualitative variables were represented by counts and weighted percentages, with their proportions analyzed using logistic regression.

A weighted multivariable logistic regression model was applied to research the link between healthy lifestyle scores and the risk of infertility. Table S3 shows that all variance inflation factors (VIFs) were well below the multicollinearity threshold (VIF = 10). Multivariable model 1 controlled for race/ethnicity (non-Hispanic white, others) and age (< 30, ≥ 30 years). Model 2 additionally adjusted for marital status (married, others), family PIR (< 3.5, ≥ 3.5), education level (below high school, high school and above), hypertension (yes, no), and diabetes (yes, no). Additionally, multivariable-adjusted odds ratios (OR) with 95% confidence intervals (CI) were assessed for infertility with each additional healthy lifestyle factor. Stratified and multiplicative interaction analyses involving all covariates were conducted to verify the consistency of the results. Multiplicative interactions were evaluated through likelihood ratio tests. The relative excess risk due to interaction (RERI) was also calculated to assess interaction from an additive perspective [31]. To evaluate how lifestyle factors influence infertility, we first examined the relationship between each lifestyle factor and infertility. In this analysis, we adjusted for all other factors. Next, we generated five new healthy lifestyle scores by excluding one lifestyle factor at a time. We then analyzed how these scores were associated with infertility, while adjusting for the excluded factor.

Sensitivity analyses were organized to assess the reliability of the results. First, women with a reported history of hysterectomy or ovariectomy were removed. Second, the optimal level of alcohol consumption was redefined as none or low-to-moderate. Third, propensity score (PS) adjustment was applied to address observed confounding. Fourth, multiple imputation (MI) was employed to address the missing covariate data. Fifth, we evaluated potential residual confounding using E-values. E-values indicate the minimum connection an unmeasured confounder must have with both the exposure and outcome, considering the measured covariate [32, 33]. Finally, a weighted healthy lifestyle score was developed to more accurately represent the influence of each lifestyle factor on the outcome [34]. This weighted standardized score was computed using β coefficients from a logistic regression model that included all five lifestyle factors and adjusted for all covariates. To calculate the score, we multiplied each binary lifestyle factor by its corresponding β coefficient. We then summed these products, divided by the total of the β coefficients, and multiplied by 5. This process resulted in a score ranging from 0 to 5, which reflects the adjusted odds ratios for each lifestyle factor within the combination of five factors. To reduce the effect of extreme groups, the scores were categorized into quartiles, and a restricted cubic spline (RCS) with three knots was plotted to illustrate the correlation between the weighted healthy lifestyle score and infertility in a dose-response manner.

All statistical analyses were conducted using R version 4.3.2 (The R Foundation for Statistical Computing, Vienna, Austria). Two-sided P-values < 0.05 were deemed statistically significant.

## Results

### Population characteristics

This cross-sectional study included 2,154 non-pregnant women of reproductive age, with a weighted mean age of 32.05 years, and 56.62% of participants being married (Table 1). The prevalence rates for low-to-moderate alcohol drinking, current nonsmoking, adequate physical activity, optimal waist circumference, and healthy diet were 82.97%, 77.77%, 45.88%, 22.42%, and 42.49%, respectively (Table 1). Population characteristics based on healthy lifestyle scores are presented in Table S4. Females with a greater number of healthy lifestyle factors tended to be younger, belong to other races/ethnicities, be married, have higher educational levels, enjoy better income status, and exhibit higher HEI-2015 scores, as well as lower BMI and waist circumference values (Table S4). Additionally, women with fewer healthy lifestyle factors had a higher likelihood of being hypertensive and diabetic (Table S4).

**Table 1:** Characteristics of study participants

Characteristics^a^	Overall (*N* = 2154)
Age, years	32.05 (0.24)
BMI, kg/m^2^	29.47 (0.28)
Waist circumference, cm	95.92 (0.66)
HEI-2015	52.54 (0.58)
Race/ethnicity, *n* (%)	
Non-Hispanic white	809 (60.36)
Non-Hispanic black	467 (12.65)
Mexican American	328 (10.37)
Others	550 (16.62)
Marital status, *n* (%)	
Married	1279 (56.62)
Others	875 (43.38)
Education attainment, *n* (%)	
Under high school	264 (9.13)
High school	409 (18.93)
Above high school	1481 (71.94)
Family PIR, *n* (%)	
< 1.3	723 (26.46)
1.3-<3.5	826 (37.22)
≥ 3.5	605 (36.31)
Current nonsmoking, *n* (%)	1691 (77.77)
Low-to-moderate alcohol drinking, *n* (%)	1784 (82.97)
Adequate physical activity, *n* (%)	891 (45.88)
Healthy diet, *n* (%)	862 (42.49)
Optimal waist circumference, *n* (%)	473 (22.42)
No. of healthy lifestyle factors, *n* (%)	
0	55 (2.77)
1	300 (13.09)
2	623 (27.12)
3	657 (30.20)
4	411 (20.46)
5	108 (6.35)
Hypertension, *n* (%)	363 (14.93)
Diabetes, *n* (%)	158 (6.22)

^a^ Continuous variables were represented as weighted means with standard errors and categorical variables as counts and weighted percentages, respectively. The total percentages may not equal 100% due to rounding of decimals and the presence of missing values

Abbreviations: BMI, body mass index; HEI, Healthy Eating Index; PIR, poverty-income ratio

### Association between healthy lifestyle score and infertility

In the crude model, the OR (95% CIs) for infertility among participants with 4–5 healthy lifestyle factors compared to those with 0–1 was calculated to be 0.38 (95% CI: 0.22–0.68). After controlling for all covariates, women exhibiting 4 to 5 healthy lifestyle factors demonstrated a 59% decrease in infertility risk (OR: 0.41, 95% CI: 0.23–0.76) relative to those with 0 to 1 healthy lifestyle factors (Table 2). Additionally, each increment in healthy lifestyle factors corresponded to a 21% decline in infertility risk (OR: 0.79, 95% CI: 0.68–0.92) (Table 2).

**Table 2:** Association of healthy lifestyle score with risk of infertility

Variable	No. of healthy lifestyle factors				Each additional healthy lifestyle factor
	0–1	2	3	4–5	
Case/total (%)	63/355 (17.75)	68/623 (10.91)	78/657 (11.87)	42/519 (8.09)	251/2154 (11.65)
Crude model	1.00 (reference)	0.51 (0.29–0.92)	0.66 (0.45–0.96)	0.38 (0.22–0.68)	0.78 (0.68–0.89)
Model 1^a^	1.00 (reference)	0.52 (0.28–0.95)	0.67 (0.45-1.00)	0.41 (0.23–0.75)	0.80 (0.69–0.92)
Model 2^b^	1.00 (reference)	0.48 (0.26–0.87)	0.64 (0.44–0.94)	0.41 (0.23–0.76)	0.79 (0.68–0.92)

^a^ Model 1 was adjusted for age (< 30, ≥ 30 years) and race/ethnicity (non-Hispanic white, others)

^b^ Model 2 was further adjusted for marital status (married, others), family poverty-income ratio (< 3.5, ≥ 3.5), education attainment (below high school, high school and above), hypertension (yes, no), and diabetes (yes, no)

### Stratified, interaction, and sensitivity analyses

To assess whether the bond between the healthy lifestyle score and the risk of infertility varied by race/ethnicity, age, marital status, family PIR, education attainment, hypertension, and diabetes, stratified and interaction analyses were conducted (Table S5). The negative correlation between the healthy lifestyle score and infertility appeared to be more pronounced in individuals aged under 30 years (P-interaction = 0.03). After controlling for all covariates, each additional healthy lifestyle factor was associated with a 38% reduction in infertility risk (OR: 0.62, 95% CI: 0.44–0.89) among women under 30 years old, and a 15% reduction in infertility risk (OR: 0.85, 95% CI: 0.72–1.01) among women aged 30 and older. However, no evidence of interaction on the additive scale was identified (Table S6).

Among the individual lifestyle factors examined, only optimal waist circumference was significantly associated with a reduction of infertility risk, with an OR (95% CIs) of 0.40 (95% CI: 0.24–0.65) (Table S7). When any single factor from the healthy lifestyles score was excluded, the association between the four-component lifestyle score and infertility weakened. Specifically, after excluding low-to-moderate alcohol consumption, current nonsmoking, adequate physical activity, optimal waist circumference, and healthy diet, the ORs (95% CIs) for individuals with 3–4 healthy lifestyle factors compared to those with 0–1 were as follows: 0.66 (95% CI: 0.40–1.10), 0.64 (95% CI: 0.38–1.09), 0.55 (95% CI: 0.34–0.88), 0.68 (95% CI: 0.45–1.01), and 0.54 (95% CI: 0.35–0.83), respectively (Table S8).

**Fig. 1:**
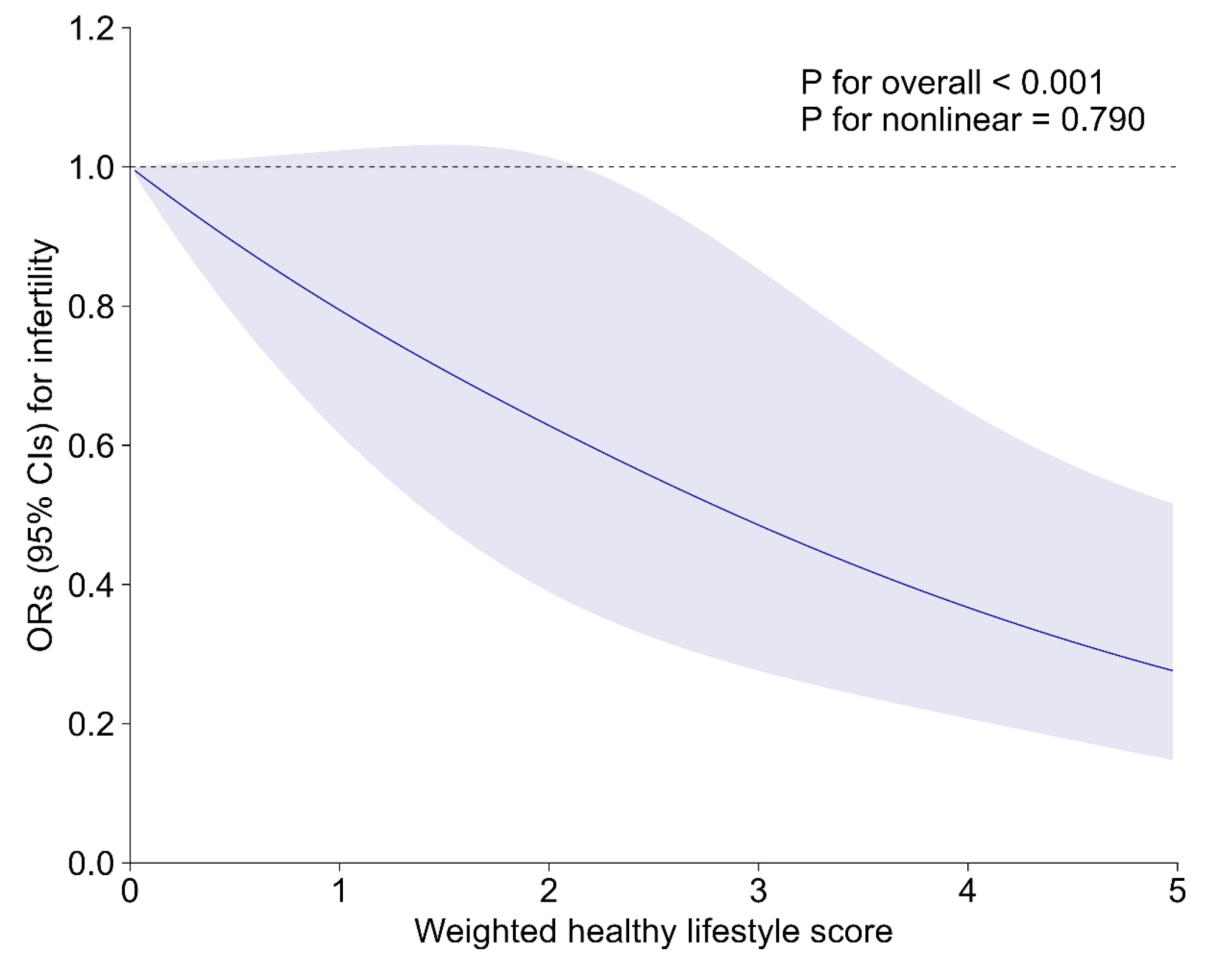
Association of weighted healthy lifestyle score with risk of infertility. The line represents multivariable-adjusted OR, and the shaded area represents 95% CI. Model was adjusted for age (< 30, ≥ 30 years), race/ethnicity (non-Hispanic white, others), marital status (married, others), family poverty-income ratio (< 3.5, ≥ 3.5), education attainment (below high school, high school and above), hypertension (yes, no), and diabetes (yes, no). Abbreviations: CI, confidence interval; OR, odds ratio

The sensitivity analyses are detailed in Tables S9-S15. The interplay between the healthy lifestyle score and the likelihood of infertility remained largely unchanged even after excluding women with a history of ovariectomy or hysterectomy, redefining healthy alcohol consumption levels, adjusting for propensity scores, and imputing missing covariates using multiple imputations (Table S9 – S12). The E-value was 4.31 (Table S13), indicating that a very strong confounder would be needed to negate the negative correlation between the healthy lifestyle score and infertility risk. A weighted healthy lifestyle score was ultimately developed. As indicated in Table S14, optimal waist circumference had the greatest contribution to the weighted healthy lifestyle score (weighted β = 0.56), followed by current nonsmoking (weighted β = 0.21), low-to-moderate alcohol consumption (weighted β = 0.21), adequate physical activity (weighted β = 0.03), and a healthy diet (weighted β = 0.00). After adjusting for covariates, women in the top quartile of the weighted score had a 65% lower infertility risk than those in the bottom quartile (OR: 0.35, 95% CI: 0.20–0.61) (Table S15). A linear inverse dose-response correlation between the weighted healthy lifestyle score and infertility was identified in the RCS analysis (P-overall < 0.001, P-nonlinearity = 0.790) (Fig. 1).

## Discussion

In this cross-sectional research involving women of reproductive age, greater adherence to healthy lifestyle factors, including healthy diet, optimal waist circumference, current nonsmoking, adequate physical activity, and low-to-moderate alcohol drinking, was associated with a lower infertility risk. The likelihood of infertility was reduced by 21% with each increment in healthy lifestyle factors. Furthermore, women who embraced 4 or more healthy lifestyle factors experienced a 59% reduction in the risk of infertility. This inverse association was pronounced in females under 30 years of age. Additionally, optimal waist circumference is the foremost factor contributing to this inverse relationship.

To date, many studies have assessed the connection between individual lifestyle factors and infertility risk, including body composition, alcohol consumption, smoking, dietary patterns, and physical activity [9, 13, 16, 35, 36]. However, research examining the combined effects of multiple lifestyle factors on infertility is limited. The Nurses’ Health Study II examined how lifestyle affects infertility due to ovulatory disorders and indicated that most infertility cases linked to these disorders could be preventable with a “fertility diet,” maintaining a healthy weight and exercising regularly [21]. Additionally, another cohort study found that lifestyle factors such as smoking, social deprivation score, alcohol consumption, age, coffee or tea intake, and weight significantly and cumulatively affect fecundity, with couples having more than four negative lifestyle variables experiencing a sevenfold increase in time to pregnancy [37]. Our study is the first to examine the link between combined healthy lifestyles and infertility risk, underscoring the potential advantages of a holistic lifestyle management strategy for addressing infertility in reproductive-age women. The government might consider enhancing educational efforts for women of reproductive age to promote the benefits of healthy lifestyles for reproductive health.

Age is a well-established factor affecting a woman’s fertility, with the likelihood of pregnancy remaining stable from puberty until around age 30, after which it declines rapidly until menopause [38, 39]. Our analysis indicates that among women under 30, healthy lifestyle factors have a strong inverse association with infertility. Before the age of 30, fertility is generally stable, making healthy lifestyle choices crucial for maintaining this advantage [39, 40]. Unhealthy habits during this time can disrupt fertility. In contrast, women over 30 face challenges such as declining ovarian reserve and increased reproductive health issues, including endometriosis, endometrial polyps, tubal disease, and uterine fibroids, all of which significantly contribute to infertility and may reduce the effectiveness of healthy lifestyle factors [41–43]. Additionally, younger women under 30 are frequently affected by academic and social pressures, which, coupled with a lack of awareness about reproductive health, can result in neglecting healthy lifestyle choices [44]. Providing education and guidance on healthy lifestyle choices to women under 30 can enhance fertility outcomes significantly. This suggests that it may be beneficial for infertile couples under 30 seeking hospital treatment to consider making lifestyle changes, along with receiving education on basic pregnancy knowledge, rather than immediately starting assisted reproductive treatments.

Our research indicates that optimal waist circumference is the most closely linked factor to infertility among combined healthy lifestyles, likely because it reflects overall health status and metabolic condition. Waist circumference is an indicator of obesity and is associated with visceral fat and abdominal obesity [45]. The mechanisms by which visceral fat accumulation contributes to infertility are intricate and multifactorial. Excess fat accumulation often causes abnormal blood vessel growth in adipose tissue, resulting in local hypoxia and triggering insulin resistance [46]. Meanwhile, abdominal fat accumulation can activate the sympathetic nervous system, leading to abnormal secretion of adipokines like leptin, which contributes to insulin resistance and promotes chronic inflammation [47, 48]. Leptin influences kisspeptin neuron activity, indirectly regulating gonadotropin-releasing hormone and affecting the hypothalamic-pituitary-ovarian axis, which in turn impacts ovulation and uterine function, potentially leading to infertility [49]. Insulin resistance can result in elevated insulin and insulin-like growth factor-1 levels, which excessively stimulate androgen secretion, disrupt normal follicular development and ovulation, and may ultimately lead to infertility [50, 51]. Additionally, chronic low-grade inflammation increases pro-inflammatory cytokines like TNF-α and IL-6, which can damage the uterine microenvironment and impair ovarian function, contributing to infertility [49, 52]. Furthermore, excess fat can raise luteinizing hormone levels and disrupt the androgen-to-estrogen ratio, altering the endocrine environment and negatively impacting follicle development and atresia, leading to infertility [53, 54]. However, the precise mechanisms involved require further investigation.

High-quality data and a representative database are notable strengths of the current study. Additionally, comprehensive sensitivity analyses were performed to confirm the stability of our results. However, several limitations need to be recognized. First, the cross-sectional nature of this study restricts the establishment of causality between infertility risk and combined healthy lifestyles. Future longitudinal studies are essential to explore this cause-and-effect relationship. Second, some information was gathered using self-reported surveys, which may result in potential bias (e.g., recall bias or social desirability bias); however, the involvement of trained professionals in conducting the interviews enhances the reliability of the data. Despite these efforts, residual bias may still exist, and this should be considered when interpreting the results. Third, the dataset is obtained from a national survey conducted in the US, and it is uncertain whether the findings apply to other ethnic groups. Finally, the database lacks information on the causes of infertility, limiting our analysis to a broad overview and preventing us from conducting more targeted assessments based on infertility causes.

## Conclusion

In conclusion, our study emphasizes the association between adherence to healthy lifestyles and a reduced risk of infertility. These findings underscore the importance of thorough lifestyle interventions for individuals of reproductive age, especially women under 30, with a focus on achieving optimal waist circumference. Public health initiatives could consider enhancing access to healthy diets, physical activity, and resources to reduce alcohol consumption and smoking for reproductive-aged women. Additionally, it may be beneficial to emphasize the role of combined healthy lifestyle management in infertility treatment guidelines. However, higher-quality cohort studies are needed to confirm our findings and the mechanisms require further investigation.

## Electronic supplementary material

Below is the link to the electronic supplementary material.

Supplementary Material 1

## Data Availability

The datasets supporting this article’s conclusions can be accessed through NHANES at https://www.cdc.gov/nchs/nhanes/.
