# Supplementary material for: Association between combined healthy lifestyles and infertility: a cross-sectional study in US reproductive-aged women
Source: BMC Public Health. 2025 Jan 14;25:153. doi: 10.1186/s12889-025-21395-2 (PMC11734407; doi:10.1186/s12889-025-21395-2)
Supplement: Supplementary file 1 — Supplementary Material 1 [file 12889_2025_21395_MOESM1_ESM.docx]

**Supplementary Materials 1**

**Association between combined healthy lifestyles and infertility: a cross-section study in US reproductive-aged women**

**Table of Contents**

[Table S1. Missing number and proportions of covariates. 3](#_Toc177655814)

[Table S2. Definitions of healthy lifestyle factors for women in this study. 4](#_Toc177655815)

[Table S3. Variance inflation factors of variables in multivariable logistic regression models. 5](#_Toc177655816)

[Table S4. Characteristics of participants with different healthy lifestyle scores from the National Health and Nutrition Examination Survey. 6](#_Toc177655817)

[Table S5. Subgroup analysis of the association between healthy lifestyle score and infertility 8](#_Toc177655818)

[Table S6. Combined effects of healthy lifestyle score and confounders on infertility. 9](#_Toc177655819)

[Table S7. Associations of individual healthy lifestyle factors with risk of infertility. 11](#_Toc177655820)

[Table S8. Associations of different lifestyle scores consisting of four lifestyle scores with risk of infertility. 12](#_Toc177655821)

[Table S9. Association of healthy lifestyle score with risk of infertility after excluding women who reported a history of ovariectomy or hysterectomy. 13](#_Toc177655822)

[Table S10. Association of healthy lifestyle score with risk of infertility after redefining the healthy level of alcohol drinking. 14](#_Toc177655823)

[Table S11. Association of healthy lifestyle score with risk of infertility after propensity score adjustment. 15](#_Toc177655824)

[Table S12. Association of healthy lifestyle score with risk of infertility after imputing missing covariates with multiple imputations. 16](#_Toc177655825)

[Table S13. E-values and lower limit of 95% CIs for the association of healthy lifestyle score with risk of infertility. 17](#_Toc177655826)

[Table S14. The coefficients of each healthy lifestyle factor in the logistic regression model. 18](#_Toc177655827)

[Table S15. Association of weighted healthy lifestyle score with risk of infertility. 19](#_Toc177655828)

[Figure S1. Flow of eligible participants selection. 20](#_Toc177655829)

# Table S1. Missing number and proportions of covariates.

| **Covariates** | **No. of missing** | **Missing proportion (%)** |
| --- | --- | --- |
| Age | 0 | 0 |
| Sex | 0 | 0 |
| Race/ethnicity | 0 | 0 |
| Family PIR | 159 | 6.87 |
| Education attainment | 0 | 0 |
| Hypertension | 0 | 0 |
| Diabetes | 0 | 0 |

Abbreviation: PIR, poverty-income ratio.

# Table S2. Definitions of healthy lifestyle factors for women in this study.

| **Factor** | **Healthy level** | **Unhealthy level** |
| --- | --- | --- |
| Tobacco smoking | Current nonsmoking | Current smoking |
| Alcohol drinking | 1-14 g/day | 0 or >14 g/day |
| Physical activity | Moderate-to-vigorous leisure-time physical activity of ≥150 min/week | Moderate-to-vigorous leisure-time physical activity of <150 min/week |
| Diet | Top two-fifths of HEI-2015 score^a^ | Bottom three-fifths of HEI-2015 score^a^ |
| Waist circumference | Waist circumference <80 cm | Waist circumference ≥80 cm |

^a^ The components of the HEI-2015 score included intakes of total vegetables, greens and beans, total and whole fruits, whole grains, refined grains, dairy, total protein foods, seafood and plant proteins, fatty acids, saturated fats, sodium and added sugars

Abbreviation: HEI, Healthy Eating Index.

# Table S3. Variance inflation factors of variables in multivariable logistic regression models.

| **Variable** | **VIF** |
| --- | --- |
| Age | 1.67 |
| Race/ethnicity | 1.30 |
| Marital status | 1.46 |
| Family PIR | 1.55 |
| Education attainment | 1.37 |
| Hypertension | 1.31 |
| Diabetes | 1.23 |
| Healthy lifestyle score | 1.88 |

Abbreviations: PIR, poverty-income ratio; VIF, variance inflation factor.

# Table S4. Characteristics of participants with different healthy lifestyle scores from the National Health and Nutrition Examination Survey.

| **Characteristics^a^** | **No. of healthy lifestyle factors** | | | | ***P*-value^b^** |
| --- | --- | --- | --- | --- | --- |
|  | **0-1** | **2** | **3** | **4-5** |  |
| No. of participants | 355 | 623 | 657 | 519 |  |
| Age, years | 32.83 (0.44) | 32.40 (0.37) | 32.46 (0.41) | 30.77 (0.44) | <0.001 |
| BMI, kg/m^2^ | 32.62 (0.50) | 31.69 (0.36) | 29.41 (0.41) | 25.42 (0.38) | <0.001 |
| Waist circumference, cm | 104.69 (1.16) | 101.46 (0.87) | 95.64 (0.81) | 85.44 (0.88) | <0.001 |
| HEI-2015 | 42.03 (0.54) | 45.78 (0.48) | 53.58 (0.72) | 64.41 (0.52) | <0.001 |
| Race/ethnicity, n (%) |  |  |  |  | 0.002 |
| Non-Hispanic white | 160 (63.19) | 215 (55.66) | 227 (58.06) | 207 (66.01) |  |
| Non-Hispanic black | 91 (14.96) | 162 (15.42) | 138 (13.17) | 76 (7.91) |  |
| Mexican American | 45 (8.65) | 98 (11.61) | 128 (13.03) | 57 (7.14) |  |
| Others | 59 (13.20) | 148 (17.31) | 164 (15.74) | 179 (18.94) |  |
| Marital status, n (%) |  |  |  |  | 0.002 |
| Married | 112 (31.57) | 238 (43.34) | 290 (47.71) | 235 (45.51) |  |
| Others | 243 (68.43) | 385 (56.66) | 367 (52.29) | 284 (54.49) |  |
| Education attainment, n (%) |  |  |  |  | <0.001 |
| Under high school | 77 (19.16) | 93 (12.62) | 73 (6.91) | 21 (2.16) |  |
| High school | 107 (32.83) | 132 (21.72) | 110 (16.19) | 60 (10.95) |  |
| Above high school | 171 (48.01) | 398 (65.66) | 474 (76.90) | 438 (86.89) |  |
| Family PIR, n (%) |  |  |  |  | <0.001 |
| <1.3 | 197 (47.40) | 240 (31.08) | 186 (22.28) | 100 (14.12) |  |
| 1.3-<3.5 | 119 (36.77) | 260 (40.98) | 271 (39.50) | 176 (31.12) |  |
| ≥3.5 | 39 (15.84) | 123 (27.94) | 200 (38.22) | 243 (54.76) |  |
| Current nonsmoking, n (%) | 107 (27.45) | 466 (72.81) | 605 (90.39) | 513 (98.36) | <0.001 |
| Low-to-moderate alcohol drinking, n (%) | 157 (44.43) | 524 (83.61) | 600 (91.18) | 503 (95.91) | <0.001 |
| Adequate physical activity, n (%) | 13 (4.48) | 104 (18.27) | 317 (52.04) | 457 (91.38) | <0.001 |
| Healthy diet, n (%) | 11 (2.99) | 97 (15.50) | 316 (47.75) | 438 (87.25) | <0.001 |
| Optimal waist circumference, n (%) | 12 (3.17) | 55 (9.82) | 133 (18.65) | 273 (50.80) | <0.001 |
| Hypertension, n (%) | 89 (23.41) | 134 (20.15) | 94 (12.22) | 46 (7.67) | <0.001 |
| Diabetes, n (%) | 39 (9.14) | 52 (8.15) | 51 (5.98) | 16 (2.79) | 0.013 |

^a^ Continuous variables were expressed as weighted means and standard errors and categorical variables were expressed as numbers and weighted percentages. The sums of percentages may not reach 100%, owing to the rounding of decimals and missing values.

^b^ Characteristics across healthy lifestyle groups were compared with linear regression for continuous variables and logistic regression for categorical variables.

Abbreviations: BMI, body mass index; HEI, Healthy Eating Index; PIR, poverty-income ratio.

# Table S5. Subgroup analysis of the association between healthy lifestyle score and infertility.

| **Confounders** | **No. of subjects** | **OR (95% CI)^a^** | ***P*-interaction** |
| --- | --- | --- | --- |
| **Overall** | 2154 | 0.79 (0.68-0.92) |  |
| **Age, years** |  |  | **0.03** |
| <30 | 823 | 0.62 (0.44-0.89) |  |
| ≥30 | 1331 | 0.85 (0.72-1.01) |  |
| **Race/ethnicity** |  |  | 0.74 |
| Others | 1345 | 0.75 (0.60-0.93) |  |
| Non-Hispanic white | 809 | 0.82 (0.67-1.00) |  |
| **Marital status** |  |  | 0.63 |
| Others | 1279 | 0.76 (0.61-0.94) |  |
| Married | 875 | 0.81 (0.66-0.98) |  |
| **Family PIR** |  |  | 0.13 |
| <3.5 | 1549 | 0.71 (0.57-0.88) |  |
| ≥3.5 | 605 | 0.95 (0.75-1.20) |  |
| **Education attainment** |  |  | 0.95 |
| High school and below | 673 | 0.77 (0.56-1.06) |  |
| Above high school | 1481 | 0.81 (0.66-1.00) |  |
| **Hypertension** |  |  | 0.57 |
| No | 1791 | 0.78 (0.67-0.91) |  |
| Yes | 363 | 0.83 (0.56-1.22) |  |
| **Diabetes** |  |  | 0.91 |
| No | 1996 | 0.80 (0.69-0.92) |  |
| Yes | 158 | 0.63 (0.39-1.02) |  |

^a^ Data were presented as odds ratio (95% confidence interval) associated with each additional healthy lifestyle factor. Models were adjusted for age (<30, ≥30 years), race/ethnicity (non-Hispanic white, others), marital status (married, others), family poverty-income ratio (<3.5, ≥3.5), education attainment (above high school, high school and below), hypertension (yes, no), and diabetes (yes, no).

Abbreviations: CI, confidence interval; OR, odds ratio.

# Table S6. Combined effects of healthy lifestyle score and confounders on infertility.

| **Confounders** | **Healthy lifestyle score** | **No. of participants** | **OR (95% CI)^a^** | **RERI (95% CI)** |
| --- | --- | --- | --- | --- |
| **Age, years** |  |  |  | 0.51 (–0.27, 1.30) |
| ≥30 | 0-3 | 1055 | 1.00 (reference) |  |
| ≥30 | 4-5 | 276 | 0.86 (0.48-1.53) |  |
| <30 | 0-3 | 580 | 0.67 (0.46-0.97) |  |
| <30 | 4-5 | 243 | 0.18 (0.08-0.43) |  |
| **Race/ethnicity** |  |  |  | 0.00 (–0.57, 0.58) |
| Non-Hispanic white | 0-3 | 602 | 1.00 (reference) |  |
| Non-Hispanic white | 4-5 | 207 | 0.67 (0.35-1.26) |  |
| Others | 0-3 | 1033 | 0.89 (0.63-1.28) |  |
| Others | 4-5 | 312 | 0.56 (0.28-1.09) |  |
| **Marital status** |  |  |  | –0.33 (–1.31, 0.66) |
| Others | 0-3 | 995 | 1.00 (reference) |  |
| Others | 4-5 | 284 | 0.61 (0.31-1.22) |  |
| Married | 0-3 | 640 | 2.20 (1.50-3.22) |  |
| Married | 4-5 | 235 | 1.48 (0.80-2.75) |  |
| **Family PIR** |  |  |  | 0.02 (–0.47, 0.51) |
| <3.5 | 0-3 | 1273 | 1.00 (reference) |  |
| <3.5 | 4-5 | 276 | 0.67 (0.35-1.26) |  |
| ≥3.5 | 0-3 | 362 | 0.86 (0.52-1.40) |  |
| ≥3.5 | 4-5 | 243 | 0.55 (0.32-0.92) |  |
| **Education attainment** |  |  |  | –0.08 (–0.99, 0.82) |
| High school and below | 0-3 | 592 | 1.00 (reference) |  |
| High school and below | 4-5 | 81 | 0.79 (0.28-2.24) |  |
| Above high school | 0-3 | 1043 | 0.80 (0.55-1.17) |  |
| Above high school | 4-5 | 438 | 0.50 (0.25-1.00) |  |
| **Hypertension** |  |  |  | 0.40 (–1.17, 1.96) |
| Yes | 0-3 | 317 | 1.00 (reference) |  |
| Yes | 4-5 | 46 | 1.00 (0.28-3.63) |  |
| No | 0-3 | 1318 | 0.79 (0.48-1.29) |  |
| No | 4-5 | 473 | 0.48 (0.25-0.90) |  |
| **Diabetes** |  |  |  | 0.07 (–1.97, 2.12) |
| Yes | 0-3 | 142 | 1.00 (reference) |  |
| Yes | 4-5 | 16 | 0.80 (0.12-5.27) |  |
| No | 0-3 | 1493 | 0.71 (0.43-1.19) |  |
| No | 4-5 | 503 | 0.46 (0.22-0.96) |  |

^a^ Models were adjusted for age (<30, ≥30 years), race/ethnicity (non-Hispanic white, others), marital status (married, others), family poverty-income ratio (<3.5, ≥3.5), education attainment (above high school, high school and below), hypertension (yes, no), and diabetes (yes, no).

Abbreviations: CI, confidence interval; OR, odds ratio; RERI, relative excess risk due to interaction.

# Table S7. Associations of individual healthy lifestyle factors with risk of infertility.

| **Healthy lifestyle factor** | **OR (95% CI)** |
| --- | --- |
| Current nonsmoking | 0.71 (0.48-1.05) |
| Low-to-moderate drinking | 0.70 (0.48-1.03) |
| Adequate physical activity | 0.96 (0.67-1.37) |
| Healthy diet | 1.00 (0.67-1.50) |
| Optimal waist circumference | 0.40 (0.24-0.65) |

Data were presented as odds ratio (95% confidence interval). Covariates included in models were shown in the footnote of Table 2, and five lifestyle factors were mutually adjusted for each other.

# Table S8. Associations of different lifestyle scores consisting of four lifestyle scores with risk of infertility.

| **Score** | **No. of healthy lifestyle factors** | | |
| --- | --- | --- | --- |
|  | **0-1** | **2** | **3-4** |
| Score consisting of smoking, alcohol consumption, physical activity, and waist circumference | 1.00 (reference) | 0.57 (0.38-0.84) | 0.54 (0.35-0.83) |
| Score consisting of alcohol consumption, physical activity, diet, and waist circumference | 1.00 (reference) | 1.02 (0.74-1.39) | 0.64 (0.38-1.09) |
| Score consisting of smoking, physical activity, diet, and waist circumference | 1.00 (reference) | 1.06 (0.78-1.46) | 0.66 (0.40-1.10) |
| Score consisting of smoking, alcohol consumption, diet, and waist circumference | 1.00 (reference) | 0.57 (0.37-0.86) | 0.55 (0.34-0.88) |
| Score consisting of smoking, alcohol consumption, physical activity, and diet | 1.00 (reference) | 0.52 (0.27-1.01) | 0.68 (0.45-1.01) |

Data were presented as odds ratio (95% confidence interval). Covariates included in models are shown in the footnote of Table 2.

# Table S9. Association of healthy lifestyle score with risk of infertility after excluding women who reported a history of ovariectomy or hysterectomy.

| **Variable** | **No. of healthy lifestyle factors** | | | | **Each additional healthy lifestyle factor** |
| --- | --- | --- | --- | --- | --- |
|  | **0-1** | **2** | **3** | **4-5** |  |
| Case/total (%) | 57/326 (17.48) | 68/587 (11.58) | 72/626 (11.5) | 42/499 (8.42) | 239/2038 (11.73) |
| Crude model | 1.00 (reference) | 0.58 (0.31-1.07) | 0.63 (0.40-0.99) | 0.42 (0.22-0.78) | 0.78 (0.67-0.92) |
| Model 1^a^ | 1.00 (reference) | 0.57 (0.31-1.08) | 0.63 (0.40-1.00) | 0.45 (0.23-0.85) | 0.80 (0.68-0.94) |
| Model 2^b^ | 1.00 (reference) | 0.54 (0.29-1.01) | 0.61 (0.40-0.94) | 0.45 (0.24-0.87) | 0.80 (0.68-0.94) |

^a^ Model 1 was adjusted for age (<30, ≥30 years) and race/ethnicity (non-Hispanic white, others).

^b^ Model 2 was further adjusted for marital status (married, others), family poverty-income ratio (<3.5, ≥3.5), education attainment (above high school, high school and below), hypertension (yes, no), and diabetes (yes, no).

# Table S10. Association of healthy lifestyle score with risk of infertility after redefining the healthy level of alcohol drinking.

| **Variable** | **No. of healthy lifestyle factors** | | | | **Each additional healthy lifestyle factor** |
| --- | --- | --- | --- | --- | --- |
|  | **0-1** | **2** | **3** | **4-5** |  |
| Case/total (%) | 44/264 (16.67) | 80/657 (12.18) | 83/682 (12.17) | 44/551 (7.99) | 251/2154 (11.65) |
| Crude model | 1.00 (reference) | 0.64 (0.35-1.19) | 0.72 (0.46-1.13) | 0.42 (0.23-0.80) | 0.78 (0.67-0.91) |
| Model 1^a^ | 1.00 (reference) | 0.65 (0.34-1.23) | 0.73 (0.46-1.17) | 0.45 (0.23-0.88) | 0.80 (0.68-0.94) |
| Model 2^b^ | 1.00 (reference) | 0.57 (0.31-1.05) | 0.67 (0.43-1.05) | 0.43 (0.22-0.85) | 0.79 (0.66-0.94) |

^a^ Model 1 was adjusted for age (<30, ≥30 years) and race/ethnicity (non-Hispanic white, others).

^b^ Model 2 was further adjusted for marital status (married, others), family poverty-income ratio (<3.5, ≥3.5), education attainment (above high school, high school and below), hypertension (yes, no), and diabetes (yes, no).

# Table S11. Association of healthy lifestyle score with risk of infertility after propensity score adjustment.

| **Variable** | **No. of healthy lifestyle factors** | | | | **Each additional healthy lifestyle factor** |
| --- | --- | --- | --- | --- | --- |
|  | **0-1** | **2** | **3** | **4-5** |  |
| Case/total (%) | 63/355 (17.75) | 68/623 (10.91) | 78/657 (11.87) | 42/519 (8.09) | 251/2154 (11.65) |
| Crude model | 1.00 (reference) | 0.51 (0.29-0.92) | 0.66 (0.45-0.96) | 0.38 (0.22-0.68) | 0.78 (0.68-0.89) |
| PS-adjusted model ^a^ | 1.00 (reference) | 0.48 (0.26-0.90) | 0.66 (0.44-0.99) | 0.42 (0.24-0.75) | 0.80 (0.69-0.92) |

^a^ PS was defined as the probability of being infertility given a set of covariates, including age (<30, ≥30 years), race/ethnicity (non-Hispanic white, others), marital status (married, others), family poverty-income ratio (<3.5, ≥3.5), education attainment (above high school, high school and below), hypertension (yes, no), and diabetes (yes, no).

Abbreviation: PS, propensity score.

# Table S12. Association of healthy lifestyle score with risk of infertility after imputing missing covariates with multiple imputations.

| **Variable** | **No. of healthy lifestyle factors** | | | | **Each additional healthy lifestyle factor** |
| --- | --- | --- | --- | --- | --- |
|  | **0-1** | **2** | **3** | **4-5** |  |
| Case/total (%) | 64/376 (17.02) | 73/672 (10.86) | 82/714 (11.48) | 42/551 (7.62) | 261/2313 (11.28) |
| Crude model | 1.00 (reference) | 0.53 (0.30-0.93) | 0.66 (0.45-0.96) | 0.38 (0.22-0.67) | 0.77 (0.67-0.89) |
| Model 1^a^ | 1.00 (reference) | 0.54 (0.30-0.97) | 0.67 (0.45-1.00) | 0.41 (0.23-0.75) | 0.79 (0.69-0.92) |
| Model 2^b^ | 1.00 (reference) | 0.48 (0.27-0.86) | 0.64 (0.45-0.92) | 0.41 (0.23-0.75) | 0.79 (0.68-0.91) |

^a^ Model 1 was adjusted for age (<30, ≥30 years) and race/ethnicity (non-Hispanic white, others).

^b^ Model 2 was further adjusted for marital status (married, others), family poverty-income ratio (<3.5, ≥3.5), education attainment (above high school, high school and below), hypertension (yes, no), and diabetes (yes, no).

# Table S13. E-values and lower limit of 95% CIs for the association of healthy lifestyle score with risk of infertility.

| **Variable** | **OR (95% CI)** | ***E*-value** |
| --- | --- | --- |
| 0-1 healthy lifestyle factors | 1.00 (ref.) | – |
| 2 healthy lifestyle factors | 0.48 (0.26-0.87) | 3.59 |
| 3 healthy lifestyle factors | 0.64 (0.44-0.94) | 2.50 |
| 4-5 healthy lifestyle factors | 0.41 (0.23-0.76) | 4.31 |
| Each additional healthy lifestyle factor | 0.79 (0.68-0.92) | 1.85 |

# Table S14. The coefficients of each healthy lifestyle factor in the logistic regression model.

| **Healthy lifestyle factor in the model** | **β coefficient** | **Weighted β coefficient** |
| --- | --- | --- |
| Tobacco smoking (healthy vs unhealthy) | –0.35 | 0.21 |
| Alcohol drinking (healthy vs unhealthy) | –0.35 | 0.21 |
| Physical activity (healthy vs unhealthy) | –0.04 | 0.03 |
| Diet (healthy vs unhealthy) | 0.00 | 0.00 |
| Waist circumference (healthy vs unhealthy) | –0.93 | 0.56 |
| Total | –1.67 | 1.00 |

# Table S15. Association of weighted healthy lifestyle score with risk of infertility.

| **Variable** | **Quartiles of weighted healthy lifestyle score** | | | | **Each additional score** |
| --- | --- | --- | --- | --- | --- |
|  | **Quartile 1 (lowest)** | **Quartile 2** | **Quartile 3** | **Quartile 4 (highest)** |  |
| Case/total (%) | 87/582 (14.95) | 73/620 (11.77) | 61/479 (12.73) | 30/473 (6.34) | 251/2154 (11.65) |
| Crude model | 1.00 (reference) | 0.80 (0.51-1.23) | 0.87 (0.57-1.32) | 0.28 (0.17-0.46) | 0.70 (0.62-0.79) |
| Model 1^a^ | 1.00 (reference) | 0.80 (0.51-1.26) | 0.90 (0.58-1.38) | 0.33 (0.20-0.56) | 0.73 (0.64-0.83) |
| Model 2^b^ | 1.00 (reference) | 0.75 (0.48-1.18) | 0.90 (0.59-1.37) | 0.35 (0.20-0.61) | 0.72 (0.62-0.83) |

^a^ Model 1 was adjusted for age (<30, ≥30 years) and race/ethnicity (non-Hispanic white, others).

^b^ Model 2 was further adjusted for marital status (married, others), family poverty-income ratio (<3.5, ≥3.5), education attainment (above high school, high school and below), hypertension (yes, no), and diabetes (yes, no).


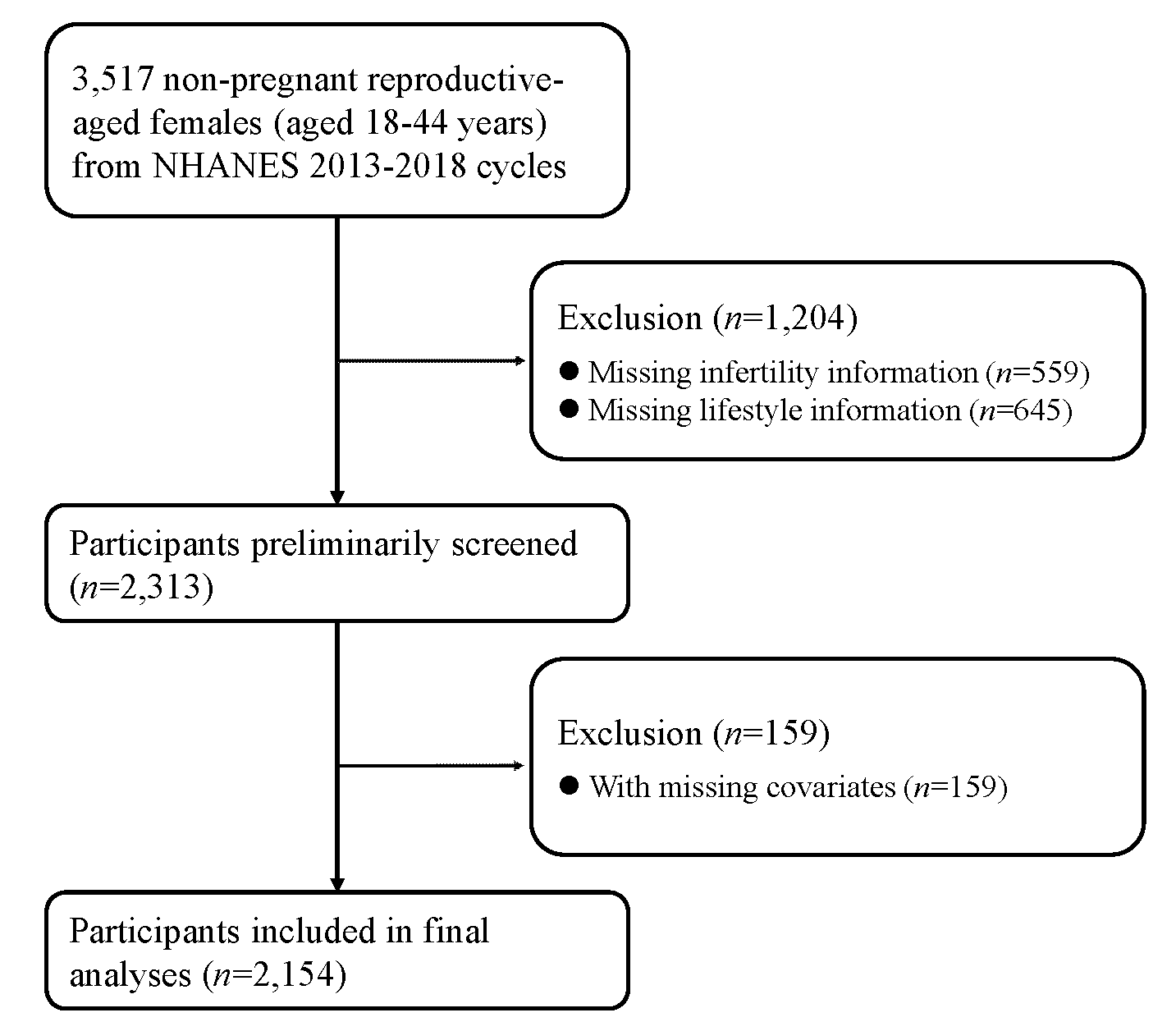


# Figure S1. Flow of eligible participants selection.

Abbreviations: NHANES, National Health and Nutrition Examination Survey.
